# Evaluation and Comparison of Physics Forceps and Conventional Forceps in Bilateral Dental Extraction: A Randomized, Split-Mouth, Clinical Study

**DOI:** 10.7759/cureus.38206

**Published:** 2023-04-27

**Authors:** Husam A Mutashar, Saif S Abdulrazaq

**Affiliations:** 1 Oral and Maxillofacial Surgery, College of Dentistry, University of Baghdad, Baghdad, IRQ

**Keywords:** anxiety during exodontia, exodontia, atraumatic tooth extraction, dental extraction, conventional forceps, physics forceps, oral surgery, dental practice, dental procedures

## Abstract

Background

Interest in atraumatic tooth extraction has increased because it aims to preserve the dental alveolus. Several tools have been designed for atraumatic extraction, including the recently invented physics forceps. This study aims to assess the physics forceps and compare the clinical outcomes to the conventional forceps.

Methodology

A prospective, randomized, split-mouth, single-blind study was conducted among 20 healthy patients needing bilateral extraction. Participants were randomly assigned to perform physics forceps extraction on one quadrant and conventional forceps extraction on the opposite quadrant. Clinical outcomes were recorded and compared, including time taken for extraction, root fracture, buccal cortical plate fracture, postoperative pain, patient satisfaction, and post-extraction socket healing.

Results

The mean extraction time of physics forceps was shorter than conventional forceps but without statistical significance. Root and buccal cortical plate fractures were lower in the physics forceps group. Statistical difference in postoperative pain was found on the third postoperative day as pain scored higher in the physics group (p = 0.038). Higher patient satisfaction was found in the physics forceps group (85%). Post-extraction socket healing was equal in 75% of the cases.

Conclusions

Physics forceps is a novel and efficient atraumatic dental extractor. It reduces intraoperative time, is associated with higher patient satisfaction, and has comparable clinical outcomes to conventional forceps.

## Introduction

Since the dawn of civilization, tooth extraction has been one of the most frequently performed surgeries. It used to be the only dental treatment performed by dentists for centuries, and several instruments have been invented for it throughout history [1].

Complete painless removal of the tooth or retained roots with minimal trauma to the underlying structures, leading to uneventful wound healing without any postoperative prosthetic complications, is the ideal outcome of a dental extraction procedure [2].

However, tooth extraction is a traumatizing event that leads to immediate apparent damage and destruction of the encompassing bone structures and soft tissues [3]. The selection of the extraction technique and instrumentation considerably affects the extent of paradental tissue destruction. Over the past decade, there has been an increased preference for atraumatic tooth extraction to preserve bone for implant insertion. Achieving the best functional, cosmetic, and orthodontic treatment outcomes relies on protecting the marginal alveolar bone crest, and to reduce damage to paradental structures, innovative extraction tools and methods have been created [4,5].

Dr. Richard Golden invented physics forceps in 2004. These forceps allow for a predictable atraumatic removal of even the most severely carious tooth with minimal or no damage to the dental alveolus. The engineering is based on the principles of the first-class lever, bone creep, and distribution of stress on the surface area without the conventional forceps methods of force application, which include squeezing, grabbing, rotating, and pulling movements [6,7].

The physics forceps consist of a handle linked to a bumper which serves as a pivot point and a beak connected to the other handle located on either the palatal or lingual side of the root. The bumper is mainly positioned within the buccal vestibule, resting on the alveolar bone. Unlike conventional forceps, there is only a single contact point on the tooth through the beak of the extractor. Furthermore, there is no squeezing force exerted on the handle or the intended tooth, but once in place, the handles are rotated by the operator’s wrist as one unit for a few degrees buccally. The internal force, or creep, builds up after 30 to 60 seconds, resulting in alveolar bone expansion and periodontal ligament (PDL) tearing. At this time, the tooth snaps out from its socket [7].

Traditional forceps consist of two first-class levers attached by a hinge. The lever’s long side is the handles of the forceps, the short side is the beaks, and the hinge serves as a fulcrum or pivot point. The handles of the forceps receive the forces applied by the operator’s hand and magnify them, allowing the forceps to grasp the tooth with considerable force. However, none of these forces is utilized to remove the tooth. Instead, increased forces may result in crown destruction [8].

Limited studies have examined physics forceps in light of patient experience, socket healing, and the common intraoperative compactions associated with tooth extraction. Extraction time, bleeding time, root fracture, buccal cortical plate fracture (BCPF), postoperative pain, patient satisfaction, and post-extraction socket healing (PSH) are the variables assessed in this study.

## Materials and methods

This prospective, randomized, controlled, split-mouth, single-blind clinical study was conducted among 20 patients who visited the Department of Oral and Maxillofacial Surgery at the College of Dentistry, University of Baghdad, seeking extraction of the same tooth bilaterally between March and September 2022. The research ethics committee of the College of Dentistry, University of Baghdad approved the study (approval ID: 391121), and written informed consent was obtained from all patients.

**Figure 1:**
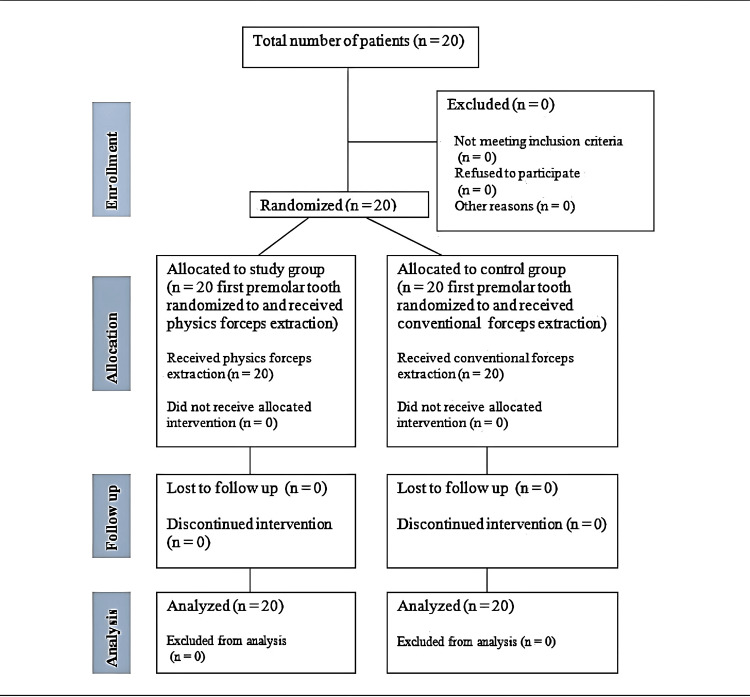
Consort diagram.

A total of 20 patients older than 10 years who required a bilateral extraction of the same teeth or teeth roots with at least 2 mm of supragingival tooth structure and were available for evaluation for seven days postoperatively were included in this study. Patients with lesions or infection in the buccal vestibular region of the tooth to be extracted or acute periapical infections, medically compromised patients with an uncontrolled systemic illness that could jeopardize extraction procedure, patients with grades II and III periodontally weak teeth, pregnant patients, and patients taking medications affecting pain assessment or who had taken any antimicrobials in the previous week were excluded from this study. All patients were randomly allocated to the control and study group using a computer-generated table of random numbers (Figure 1).

Each patient received a conventional forceps extraction on one quadrant and a physics forceps extraction on the other, with an interval of three days between the two extractions. The first author performed all extractions under rigorous aseptic settings, and the patient was blind to the type of forceps employed during each extraction. The preoperative evaluation included a thorough patient history, clinical examination, and a radiographic assessment. All patients were anesthetized with lidocaine 2% E-80 with epinephrine 1:80,000 (New Stetic S.A., Colombia) via superior alveolar and greater palatine nerve block for the upper teeth and inferior alveolar and lingual nerve block for the lower teeth.

**Figure 2:**
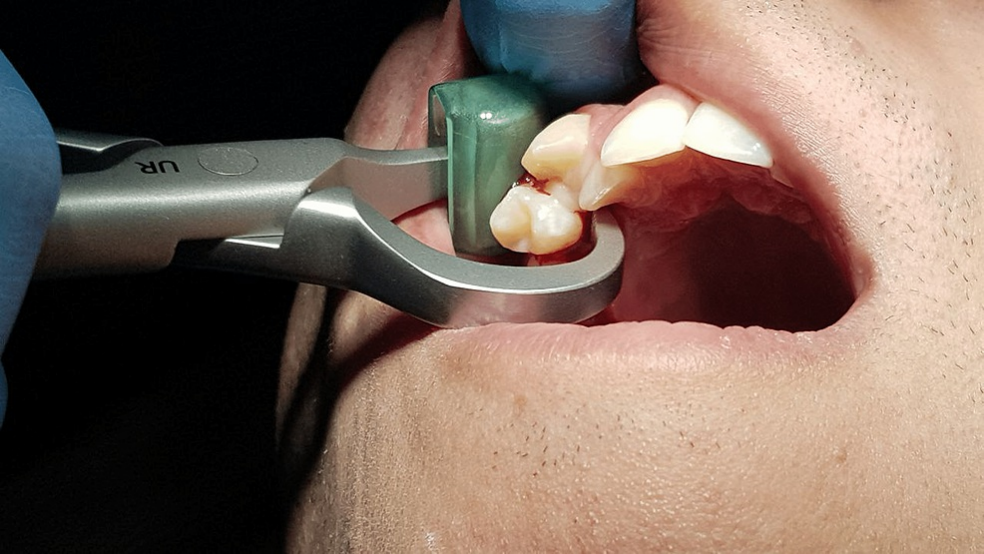
Positioning of the physics forceps during upper right first premolar extraction.

When using physics forceps, the beak was positioned on the lingual/palatal side of the tooth at or just below the cementoenamel junction. The bumper was positioned on the buccal aspect of the alveolus at the mucogingival junction. Then an uninterrupted controlled tractional force was applied until the tooth snapped out from its alveolar housing (Figure 2). In conventional forceps, the forceps were positioned as apically as possible and aligned to the tooth’s longitudinal axis after severing the fibers that connect the gingival margin to the neck of the tooth. The tooth was then extracted from the socket using torsional movements mixed with buccolingual rocking for lower teeth and gentle wiggling in a buccopalatal orientation while pulling the upper teeth.

Postoperatively, all participants were given instructions to bite on a gauze pack for approximately 30 minutes and avoid gargling and spitting for the first 24 hours. In addition, patients were instructed to maintain a soft diet and avoid eating on the extraction site with a gentle rinse for 30 seconds with chlorhexidine mouthwash 0.12% once daily starting from the second day postoperatively for one week. Analgesics were prescribed to the patients in the form of mefenamic acid 500 mg to be taken as needed. Patients were instructed to return for a follow-up appointment on the third and seventh postoperative days.

The duration of the extraction procedure was recorded in seconds using a stopwatch, starting by grasping the tooth with the forceps and ending when the tooth was removed from the socket. The BCPF integrity was carefully assessed by manual palpation along the extracted tooth socket externally and by running a dental probe on the lingual aspect of the buccal plate from inside the socket in all directions (from apical to occlusal and from mesial to distal) to check for any discontinuity or step deformity of the bone or fenestration or dehiscence-type defects, as well as by examining the extracted tooth for adherence of the buccal plate to the root surfaces. A yes/no format was used for the assessment. The root(s) fracture was assessed using the following format: one root fracture = 1, two roots fracture = 2, and three roots fracture = 3.

**Figure 3:**
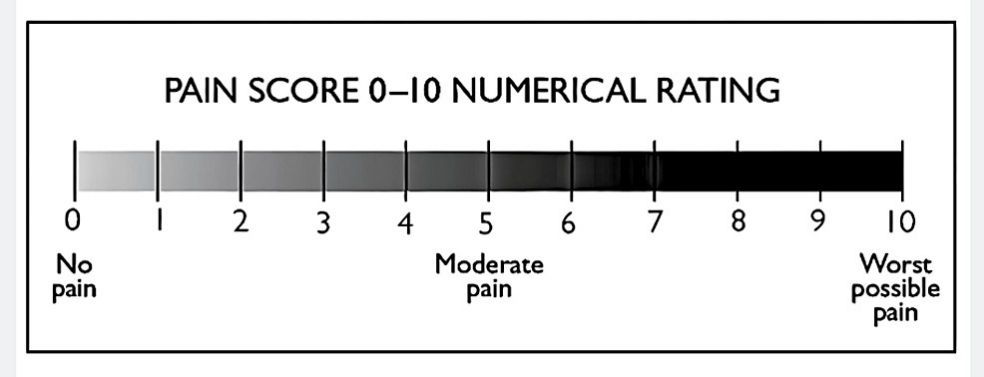
Pain numeric rating scale.

Pain scores were recorded on the evening of the operative day (D0) and on the first (D1), third (D2), and seventh (D4) postoperative days using the pain numeric rating scale (NRS), a scale from 0-10, in which 0 is no pain, and 10 is the worst Pain (Figure 3). Patient satisfaction with the extraction was measured on a 1-5-point scale, where 1 represents not satisfied, 2 is slightly satisfied, 3 is neutral, 4 is satisfied, and 5 represents very satisfied.

PSH was assessed using the Landry, Turnbull, and Howley healing index, modified by Pippi et al. [9]. For every socket, the following parameters were assessed in comparison, applying a dichotomic score (1/0) with a cumulative score of 7: (1) tissue color; pink (same color as the rest of the gingiva) or presence of redness indicating an inflammatory response; (2) granulation tissue, present or absent; (3) suppuration, present or absent; (4) swelling, present or absent; (5) epithelialization extent, partial or full; (6) tenderness on palpation, present or absent; and (7) bleeding on palpation, present or absent. Evaluation of these criteria for each site enabled the designation of healing as better, worse, or equal by comparison.

Data description, analysis, and presentation were performed using SPSS version 21 (IBM Corp., Armonk, NY, USA). Statistical analyses can be classified into the following two categories: (1) descriptive analysis, which includes minimum, maximum, mean, and standard deviation (SD) for quantitative variables, and mean rank, frequency, and percentage for qualitative variables. (2) Inferential analysis includes the Friedman test, which tests the difference for k-related mean rank Dunn Bonferroni method; the Fisher exact test, which tests the association of distribution between two qualitative variables when the expected cell counts less than 5 is >20%; the chi-square test, which tests the association of distribution between two qualitative variables when the expected cell count of less than five is <20%; the Wilcoxon sum-rank test, which is a non-parametric test for difference between the mean rank of two groups; sign test, which assesses if there is a significant difference for paired or matched observation; and repeated-measures analysis of variance.

## Results

Demographic details of the study participants are presented in Table 1.

**Table 1:** Patient’s demographics. M = male; F = female; *: p < 0.05

Variables		N	%	P-value
Age (years)	16–23	16	40	0.051*
	24–31	4	10	
Gender	M	8	20	0.503
	F	12	30	

The study sample was limited to the first premolar tooth extracted for orthodontic purposes due to the split-mouth design, and extracted teeth distribution is presented in Table 2.

**Table 2:** Distribution of the extracted teeth among groups and in the study sample. L: left; R: right; *: p < 0.05

Tooth	Groups				Fisher’s exact test	P-value	Total	
	Study		Control					
	N	%	N	%			N	%
Lower 4L	4	20	4	20	0.195	0.999*	8	20
Lower 4R	4	20	4	20			8	20
Upper 4L	6	30	6	30			12	30
Upper 4R	6	30	6	30			12	30

Extraction time was longer in the control group (51.50 ± 20.73 seconds) compared to the study group (42.85 ± 12.44 seconds); however, the difference in the mean extraction time between the groups was statistically non-significant (p = 0.118), as shown in Table 3.

**Table 3:** Descriptive and statistical test of extraction time among groups. *: p < 0.05

Groups	Extraction time in seconds		t-test	P-value
Study group	Minimum	15.00	1.60	0.118*
	Maximum	63.00		
	Mean	42.85		
	±SD	12.44		
Control group	Minimum	21.00		
	Maximum	105.00		
	Mean	51.50		
	±SD	20.73		

BCPF occurred two times (10%) in the study group and four times (20%) in the control group. While root fracture incidence was two times (10%) in the study group and three times (15%) in the control group with no statistical significance, as shown in Table 4.

**Table 4:** Distribution of BCPF and root fracture among groups. BCPF: buccal cortical plate fracture; *: p < 0.05

Variables		Groups				Statistic	P-value	Total	
		Study		Control					
		N	%	N	%			N	%
Root fracture	Yes	2	10	3	15	0.229	0.999*	5	12.5
	No	18	90	17	85			35	87.5
BCPF	Yes	2	10	4	20	0.784	0.661*	6	15.0
	No	18	90	16	80			34	85.0

As scored on D0, D1, D3, and D7 using NRS questionnaires, pain showed a significant change in each group. The fastest decline was noted in the control group, with pain scores higher in the study group, but with no significant association except that statistical hypotheses demonstrated a highly significant difference (p = 0.038) with a large effect size (0.385) on D3 (Table 5).

**Table 5:** Descriptive and statistical test of pain among groups and time (repeated-measure analysis of variance). F: Friedman test; ES: effect size; D0: operative day; D1: first postoperative day; D3: third postoperative day; D7: seventh postoperative day

Groups		D0	D1	D3	D7	F	P	ES
Study	Mean	3.150	1.900	1.050	0.000	11.941	0.00	0.499
	±SD	2.641	1.832	1.395	0.000			
Control	Mean	2.400	1.200	0.250	0.000	36.81	0.00	0.351
	±SD	2.257	1.735	0.910	0.000			
F		0.932	1.539	4.615	0	ES (r) = 0.108 large		
P-value		0.340	0.222	0.038	1			

Concerning pain parameters along different periods from D1 to D7, each group had a similar behavior that declined steadily. Statistical hypotheses demonstrated a highly significant difference regarding D1 × D3 and D1 × D7 in both groups and D2 × D7 in the study group (Table 6).

**Table 6:** Multiple pairwise comparison of pain between time by groups using BPT. MPC: multiple pairwise comparisons; BPT: Bonferroni posthoc test; D0: operative day; D1: first postoperative day; D3: third postoperative day; D7: seventh postoperative day

Group	Days	NRS, mean ± SD	Pairwise days (P-value)		
Control	D0	2.40 ± 2.257	D1	D2	D3
	D1	1.20 ± 1.735	0.102		
	D3	0.25 ± 0.910	0.012	0.954	
	D7	0.00 ± 0.000	0.000	0.518	0.999
Study	D0	3.15 ± 2.641	D1	D2	D3
	D1	1.90 ± 1.832	0.757		
	D3	1.05 ± 1.395	0.016	0.850	
	D7	0.00 ± 0.000	0.000	0.016	0.755

On comparing the degree of satisfaction between the groups, we found that the highest percentage of satisfaction was among the study group at 85% compared with 65% in the control group; however, the difference was statistically non-significant (p = 0.245) (Table 7).

**Table 7:** Distribution of patient’s satisfaction among groups. 1: not satisfied; 2: slightly satisfied; 3: neutral; 4: satisfied; 5: very satisfied; *: p < 0.05

Scores	Groups				Fisher’s exact test	P-value
	Study		Control			
	N	%	N	%		
1	1	5	0	0.00	5.018	0.245*
2	0	0.00	1	5		
3	2	10	6	30		
4	3	15	4	20		
5	14	70	9	45		

The PSH final scores on D3 were better in the study group in 5% of the cases, equal in 75% of the cases, and worse than the control group in 20% of the cases but with no significant association. However, healing on D7 was equal in both groups (Table 8).

**Table 8:** Distribution of PSH score (study to control). PSH: post-extraction socket healing; *: p < 0.05

Healing	N	%	Sign. test
Equal	15	75	0.375*
Better	1	5	
Worse	4	20	

## Discussion

Controlled forces are required for atraumatic tooth extraction to preserve the alveolar bone and gingival tissue architecture, enabling delayed or immediate dental implant insertion [6]. Different instruments and methods for atraumatic extractions have been proposed. The advantage of physics forceps over every other means of tooth extraction relates to their unique design, which can provide significant mechanical leverage by utilizing a highly efficient first-class lever [8].

In physics forceps, the torque force exerted on the alveolar bone, PDL, and the tooth is equivalent to the length of the handle (8 cm) divided by the distance between the beak and bumper (1 cm). Therefore, applying force to the bumper-attached handle increases the force on the alveolar bone, PDL, and tooth by around eight times [10].

Hyaluronidase is released when the PDL is traumatized by the forceps. Therefore, by its consistent, relentless trauma to the PDL, physics forceps generate higher amounts of hyaluronidase than conventional forceps or elevators because the forces delivered by these instruments are intermittent [8].

We implemented a split-mouth design because numerous parameters (such as nutritional status, dental hygiene, and bone health) are similar on the two sides, and the patient’s commitment was consistent. There was no operator bias because only one operator performed all extractions.

In this study, the mean extraction time in the control group was 51.50 ± 20.73 seconds, and in the study group was 42.85 ± 12.40 seconds. A shorter extraction time in the study group could be related to the unique design of physics forceps, which decreases the time frame by building up internal force within 30 to 60 seconds, facilitating bone expansion and loosening of PDL, and eventually disengaging the tooth from its socket, as opposed to the wrecking movements of conventional forceps. However, the difference was statistically non-significant. These results are in agreement with those reported by Hariharan et al. [10], who also found a shorter but non-significant difference in extraction time, as the mean extraction time of physics forceps was 29.4 seconds and conventional forceps was 43.5 seconds. Lingaraj et al. [11], Hasan [7], and Patel et al. [12] all reported a significant difference in extraction time in favor of physics forceps; however, none of these studies described the operator’s experience in using physics forceps. Physics forceps has a particular learning process because the technique of removing the tooth from its socket differs significantly from conventional forceps employed for education in dental colleges [11].

In this study, BCPF occurred two times (10%) in the study group and four times (20%) in the control group. However, the incidence was lower in the study group, and there was no statistically significant difference. Physics forceps apply a consistent and steady tractional force through the operator’s wrist movement only, reducing the possibility of buccal bone fracture. Furthermore, the buccally positioned bumper supports and maintains the buccal alveolar cortical plate in position by applying a compressive force [13]. This study is also in accordance with the previous studies that investigated BCPF in physics forceps [7,14-16]. Abdelwahab et al. [17] mentioned that the amount of force required for extracting a tooth might be determined by the shape and size of the roots. As a result, BCPF could be more prevalent when extracting multi-rooted teeth rather than single-rooted premolars and incisors.

The incidence of root fracture was two times (10%) in the study group and three times (15%) in the control group, with no statistical significance. Having a prevalence ranging from 5% to 7% and reaching 30% in teeth with curved roots, it is hardly unexpected that most investigations studied this consequence [18]. However, most studies found no difference in tooth fracture (regardless of the region) [7,12,14]. Choi et al. employed physics forceps for extracting teeth for intentional replantation (IR) and found that tip fracture occurred in 2% of the cases. They reported that physics forceps could be regarded as a reliable technique for safe and successful IR [15]. This study is also in line with the study by Shetty and John, who reported 12% root fractures in the conventional forceps group and 2% in the physics forceps group [19]. Furthermore, the lingual beak of physics forceps applies a steady and consistent tractional force along the root’s axis, and the bumper acts as the extraction fulcrum. Therefore, the conventional squeezing forces are never utilized by physics forceps; as a result, the tooth does not split, crush, or fracture.

Postoperative pain is a frequent undesirable consequence of any surgical procedure. It is multifactorial and is caused by the release of inflammatory mediators from the wounded tissue and the patient’s physiological threshold and anxiety levels [18]. The NRS was used in this study to rate the intensity of pain felt after tooth extractions, as it is easy to be understood by the patient and does not need language translation.

The mean of pain records for the control group began at a score of 2.40 on D0, reduced to 1.20 on D1, and then steadily subsided to 0.25 and 0.00 on D3 and D7, respectively. The mean of pain records in the study group began with a score of 3.15 on D0, decreased to 1.90 on D1, and then declined to 1.05 and 0.00 on D3 and D7, respectively. The results showed that pain gradually decreased significantly in both groups. On comparing pain degrees between both groups, this study found a significant difference in D3, with pain scoring higher in the study group. The reason for higher pain levels in the study group is mostly related to two factors. First, compression of buccal gingival soft tissue by the physics forceps bumper as many patients reported pain at the area of the bumper application instead of the extraction socket. Second, we did not employ an elevator before applying conventional forceps, as in conventional extraction techniques. This could have minimized surgical site trauma and pain, especially in the first few days following surgery. Kapila et al. reported a higher mean score of pain on D1 in the physics forceps group (3.04 ± 1.47) than in the conventional forceps group (2.89 ± 1.21). They also observed buccal ulcer formation in two cases in the physics forceps group on the third postoperative day caused by the excessive force exerted by the bumper on the soft tissue [20].

The healing process of the dental sockets is influenced by many local or systemic factors [21]. It is a complex process that could be separated into three overlapping phases, namely, inflammatory reaction, proliferation, and remodeling [22]. Several methodologies for assessing wound healing in oral soft tissues have been described. In this study, we used a healing index proposed by Landry, Turnbull, and Howley (1988) to describe the extent of clinical healing after periodontal surgery. It was recently modified by Pippi et al. [9] for PSH use. PSH was equal in 75% of cases on D3 and was 100% equal on D7, but it was not statistically significant on both days. These results are in accordance with Kapila et al. [20], Hariharan et al. [10], and Ramakrishna et al. [23]. Kapila et al. assessed socket healing based on the dry socket, acutely inflamed socket, and acutely infected socket; however, it remained uneventful in the two groups, as noted in D3 and D7. Ramakrishna et al. and Hariharan et al. investigated delayed healing on D1, D3, and D7 and found no evidence of delayed healing when using either forceps for regular extractions. In contrast, Basheer [24] found significantly improved healing during the first week in the physics forceps group.

Patient satisfaction is one of the key criteria used to evaluate clinical services because it is one of the most important aspects when evaluating the quality of care [25]. Patient satisfaction was measured using a five-point score system similar to the Likert scale to assess agreement or disagreement with a statement [26]. The patients’ responses were gathered at the end of the surgical procedure. A higher level of satisfaction was reported in the study group. This result could be related to two factors, namely, shorter operating time in which the patient experienced less anxiety and increased patient comfort because physics forceps applied lower pressure and force during the extraction process. These results agree with other studies that reported higher patient satisfaction and comfort among patients who received physics forceps extraction [16,27].

This study has some inherent limitations, including the small sample size and the extracted tooth type, which was limited to the first premolar tooth extracted for orthodontic reasons due to the split-mouth study design. Future research needs to be conducted with a larger sample size and including all teeth (anterior and posterior, sound and decayed) as well as root stumps.

## Conclusions

The current investigation concluded that physics forceps maintain the dental alveolus and surrounding periodontium integrity. Extractions with these forceps tend to be less disruptive than conventional forceps, require less intraoperative time, reduce the incidence of root and BCPF compared to conventional tooth extraction forceps, and are associated with higher patient satisfaction and comparable PSH. Therefore, physics forceps can be regarded as a reliable technique for atraumatic tooth extraction, which is especially important in implant and prosthetic dentistry, and a valuable addition to the tooth extraction armamentarium.
